# HCV NS3 Blocking Effect on IFN Induced ISGs Like Viperin and IL28 With and Without NS4A

**DOI:** 10.5812/hepatmon.17822

**Published:** 2014-06-08

**Authors:** Zahra Khanlari, Farzaneh Sabahi, Seyed Younes Hosseini, Mostafa Ghaderi

**Affiliations:** 1 Department of Medical Virology, Faculty of Medical Sciences, Tarbiat Modares University, Tehran, IR Iran; 2 Gastroenterohepatology Research Center, Shiraz University of Medical Sciences, Shiraz, IR Iran

**Keywords:** Hepatitis C, NS3, Interferon-Lambda Protein

## Abstract

**Background:**

Hepatitis C virus (HCV) is able to down-regulate innate immune response. It is important to know the immune pathways that this virus interacts with. HCV non-structural protein 3 (NS3) plays an important role in this viral feature. HCV NS3 protein could affect the expression of antiviral protein such as viperin, and interleukin 28whichare important proteins in antiviral response.

**Objectives:**

HCV has developed different mechanisms to maintain a persistent infection, especially by disrupting type I interferon response and subsequent suppression of expression of Interferon stimulatory genes (ISGs). Viperin, a member of ISGs, is considered as a host antiviral protein, which interferes with viral replication. Since it is a good target for some viruses to evade host responses, it is interesting to study if HCV has evolved a mechanism to interfere with this member of ISGs.

**Materials and Methods:**

We evaluated the impact of NS3, NS3/4A and a mutated nonfunctional NS3 on ISGs expression such as viperin and IL-28 after the induction of IFN signaling Jak-STAT pathway using IFN-.

**Results:**

NS3 protein disrupted the expressions of viperin gene and IL-28, an inducer for the expression of ISGs and viperin itself. By comparing the roles of NS3 and NS3/4A protease activities in suppressing the innate immune responses, we also showed that NS3 (without NS4A) has the ability to down-regulate ISGs expression, similar to that of NS3/4A.

**Conclusions:**

ISGs expression is impeded by NS3 protease activity and its interaction with Jak-STAT pathway proteins. In addition, the NS3/4A substrates spectrum seems to be similar to those of NS3.

## 1. Background

For the first time in 1989, Hepatitis C virus (HCV) was presented as the main causative agent of non-A, and non-B hepatitis (1). This virus is classified as a member of Hepacivirus genus of *Flaviviridae* family (2). Approximately 150 million people are chronically infected with HCV worldwide and more than 350000 people die every year from the disease (3).The spontaneous viral clearance usually happens only in one of five newly infected cases and this failure largely depends on HCV capacity to block interferon (IFN) type I expression and antiviral host signaling pathways (4) and other factors such as HCV genotype (5). IFNs are known as host factors which connect innate and adaptive immune responses, the mass activation of innate immune system is essential to produce an adequate response for virus neutralization (6). Type I IFNs, as effective players in innate immunity against viral infections, induce the expression of hundreds of ISGs, which ultimately regulate antiviral responses.Virus inhibitory protein, endoplasmic reticulum-associated, IFN-inducible (viperin), a newly characterized antivirus molecule is considered as a member of ISGs (7). In normal condition, the expression of viperin is low (8) but types I, II and III IFNs, double-stranded DNA and RNA, lipopolysaccharide (LPS) and viral infections can stimulate the expression of this protein (9). Both DNA and RNA viruses can stimulate overexpression of this antiviral protein, among which Japanese encephalitis virus (JEV) (10), Chikungunya virus (CHIKV) (11), yellow fever virus (12), and HCV (13) are of significant importance. As viperin is an antiviral protein, it could be used as a suitable target for silencing the primary host immune response by different viruses like JEV and CHIKV (8). In the case of HCV, viperin couldespeciallyinhibit replication of virus through interaction with lipid droplets required for efficient virion assembly (7) as well as human vesicle-associated membrane protein of 33 kDa (hVAP-33) which is present in the HCV replication complex (14). For establishment of a persistent infection, HCV is able to suppress innate antiviral mechanisms mainly by cleavage of mitochondrial antiviral signaling protein (MAVS) and Toll-interleukin-1 receptor domain-containing adaptor-inducing beta interferon (TRIF) using NS3 protease function which blocks downstream pathways. Since HCV has the ability to suppress IFN response as a frontline of antiviral defense using NS3 (2, 15, 16).We evaluated the possible effect of HCV NS3 on the expression of viperin after IFN induction and assessed its protease activity in this process. We also evaluated NS3 protein function alone or together with NS4 for its inhibitory role on the expression of viperin as well as IL-28, another member of ISGs. Altogether, our findings highlighted the fundamental role of NS3 protease activity in ISGs expression following IFN treatment and Jak-STAT stimulation and indicated the dispensable role of NS4A in protease activity of NS3.

## 2. Objectives

To investigate whether HCV NS3 protease activity down-regulates the expression of ISGs, induced by Jak-STAT pathway stimulation.

## 3. Materials and Methods

### 3.1. Cell culture

HEK293 and HepG2 cells (as a suitable model of liver immune assessment) were provided by a national cell bank (Pasteur Institute, Iran) and cultured in high glucose DMEM medium (Gibco, Germany) with 0.6 ug/mL penicillin, 60 ug/mL streptomycin, and 10% heat-inactivated FCS (Gibco, Germany) at 37℃ in a humidified incubator with 5% CO2.

### 3.2. Design of Experiment

It is known that viperin expression is induced after stimulation of type I IFN in HepG2, Huh7 and fibroblasts cells (13). Here, we compared six different treatments including 3 cell groups that were transfected with expressing plasmids 48 hours before recombinant IFN (Sigma, UK) treatment, one group was treated only by recombinant IFN alpha (1000 IU/mL) as positive control, mock group treated only with lipofectamine 2000, and the last group was a negative control group without any treatment. In addition, we used a newly approved anti-protease drug, Boceprevir (Merck Co. Canada) in 14 nm concentration to confirm our results by its protease blocking activity in all the treated groups 6 hours after IFN induction.

### 3.3. Plasmids Constructions

**Figure 1.**
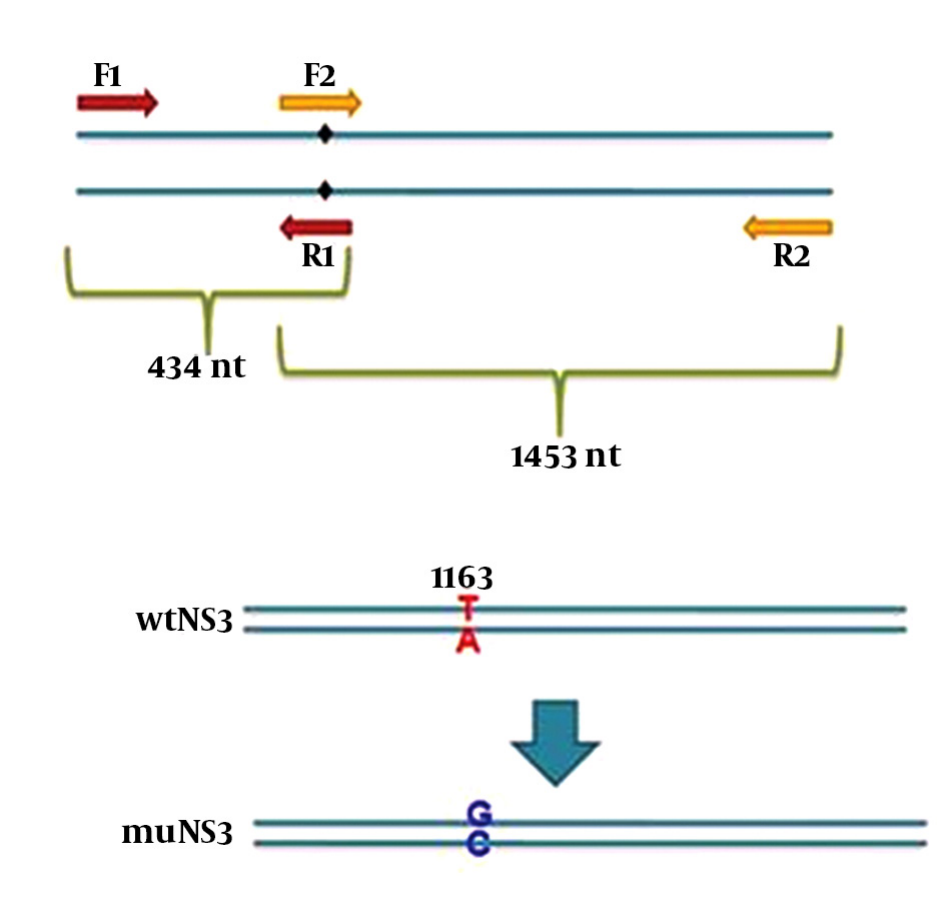
Schematic For SOEing PCR

In this study, 3 different plasmids were employed as follows: expression plasmids containing NS3/4A sequence of HCV was a kind gift from Dr. Michael M.C. Lai (Institute of Molecular Biology, Academia Sinica, Taiwan, R.O.C.), the original plasmid (gWiz) expressing wild type full length NS3 (wtNS3) of HCV with protease activity was also a kind gift from Dr. Gloria Gonzalez-Aseguinolaza (CIMA Research Center, Navarra, Spain) which was exploited as a template for the construction of a mutated NS3 gene (muNS3) at the position ser-139. Mutation at this position destroys the protease activity without altering NS3 structure (17) which is responsible for inhibition of IFN pathways, as demonstrated before. For this purpose, the nucleotide T-1163 was replaced by G (the G nucleotide highlighted bold in R1 and F2 primer sequences) in wild type NS3 sequence, using mutation harboring primers and subsequent SOEing PCR (Figure 1). At first, two PCR reactions were performed by using four modified primers F1/ R1 and F2/ R2, to amplify the left and right segments (434 bp and 1464 bp, respectively) from NS3 gene while desired point mutations appeared afterward in overlapping region. Designed primers in PCR reactions for muNS3 creation are listed in Table 1.

**Table 1.** Primer Sequences for SOEing PCR

Primer Name	Primer Sequences
**F1**	5’-ACGCGTCGACGCCACCATG-3’
**R1**	5’-CAAAGCAGTGGTCACCCGCGGAG-3’
**F2**	5’-CTCCGCGGGTGGACCACTGCTTT-3’
**R2**	5’-GCGGATATCTCAAGTGACGACCTCCAGG-3’

Underlined nucleotides were introduced into primer sequences to create new restriction sites, F2 primer sequence is complementary and reverse of R1 primer sequence and these two primers are overlapping in 23 nucleotides. PCR reactions were performed using one unit Pfu polymerase, 15 pmol of each primers and 10 ng/µL of template (linearized wtNS3 plasmid) in standard condition for 25 cycles of 60°C annealing temperature. Later, in SOEing PCR reaction, PCR products from the previous stage were mixed with equal concentrations and a PCR reaction was performed without any primer to ensure that the two amplified DNA segments were connected to each other. This reaction was performed for 12 cycles at 62°C (the annealing temperature that was defined for overlapped segments using Oligo analyzer 3.1 software. Then, the desired sequence was rescued using F1 and R2 primer pair. Afterwards, this sequence was extracted using a gel extraction kit (Qiagen, Germany). The product of this stage was digested with restriction enzymes whose cutting sites had been introduced into F1 and R2 primers end during the design (underlined in primer list). This new nucleotide modification led to the creation of a new *sacII* (Neb, New England) cutting site. The establishment of this mutation was confirmed by digesting with *sacII* restriction enzyme and sequencing, respectively. The digested product was cleaned up and then, ligated into gWiz plasmid using T4 ligase enzyme (Intron, Korea).

### 3.4. Protein Expression and Western Blotting

HepG2 cells were grown on 12-well culture plates and plasmids expressing wild or mutated NS3 and NS3/4A, transfected by using Lipofectamine 2000 (Invitrogen, Germany), then, after 48 hours, total protein was isolated using lysis buffer (150 mM sodium chloride, 1.0% NP-40, 50 mM Tris, pH 8.0). Protein concentration was determined by Nanodrop (Biowave II, Biochrom, UK) and equal amount of total protein (80 µL) was loaded in each lane of 12% SDS-PAGE. Western blotting was performed as described earlier (18). Accordingly, the membranes were introduced into 1/400 dilution of specific polyclonal antibody against NS3 (Abcam, UK), followed by incubation with horseradish peroxidase-conjugated secondary antibody (Invitrogen, Germany). Presence of antigen-antibody complex was indicated by enhanced chemiluminescence assay according to the manufacturer’s protocol (Takara, Japan) (18). Furthermore, the presence of viperin after IFN stimulation and in the presence of wtNS3, muNS3 and NS3/4A was evaluated by western blotting using 1/800 dilution of specific polyclonal antibody against viperin (Abcam, UK).

### 3.5. RNA Extraction, cDNA Synthesis and Quantitative PCR (qPCR)

Different expression plasmids containing wtNS3, muNS3 and NS3/4A were transfected into HepG2 cells. Then, 48 hours following transfection with different expression plasmids, HepG2 cells were treated with 1000 IU/mL of recombinant IFN-alpha (Sigma, UK) and 6 hours later, total RNA was extracted using Qiazol reagent (Qiagen, Germany), according to the manufacturer’s instructions. We evaluated the different concentrations of IFN-alpha at various times, and the best results were shown in 1000 IU/mL of IFN-alpha and 6 hours. The final number of cells for this purpose was approximately 2.5×10^5^ cells/ well. Reverse Transcription was performed on 300 ng total RNA, using Thermo scientific RT kit while Oligo dT was used in cDNA synthesis process. Real time PCR on the synthesized cDNA was performed using SYBR green PCR master mix (Takara, Japan) by step one ABI system (Applied Biosystem). The samples were subjected to 95°C denaturation step for 5 min, then, 40 cycles of 95°C denaturation for 50 seconds and desired annealing temperatures for 35 seconds for each primer pair. The corresponding primer pairs for IL-28, viperin, STAT1, OAS1 and GAPDH were listed in Table 2. Moreover, we used 5 pmol of each primer in real time PCR reactions. All the tests were performed in triplicate. The threshold cycle (Ct) for each specific gene, corresponding housekeeping gene (GAPDH) and their differences (∆Ct) were determined and then, submitted to formula for further semiquantification analysis, as explained elsewhere (19). All data were analyzed by using the GraphPad Prism software.

**Table 2.** Primer Sequences Used in Real-Time PCRs

Genes	Primer Sequences	Melting Temperature
**Viperin**	F: 5’-TGCTTAAGGAAGCTGGTATGGAG-3’ R: 5’-TCACCAACTTGCCCAGGTAT-3’	57 °C
**IL-28**	F: 5’-CTGCCACATAGCCCAGTTCAAGT-3’ R: 5’-ACTCTTCTAAGGCATCTTTGGCCC-3’	60 °C
**STAT1**	F: 5’-ATGTCTCAGTGGTACGAACTTCA-3’ R: 5’-TGTGCCAGGTACTGTCTATT-3’	55 °C
**OAS1**	F:5’-GATCTCAGAAATACCCCAGCCA-3’ R: 5’-AGCTACCTCGGAAGCACCTT-3’	60 °C
**GAPDH**	F:5’-ACCTGACCTGCCGTCTAGAAA-3’ R:5’-CCTGCTTCACCACCTTCTTGAT-3’	60 °C

### 3.6. Immunofluorescence

To trace NS3 protein distribution inside the cells, HEK293 cells were seeded on eight wells chamber slide (BD), then, transfected by NS3, muNS3 and NS3/4A expressing plasmids. After 48 hours, cells were fixed by ice cold acetone for 10 min, blocked with 10% goat serum for 1 hour at RT., and then, were incubated with 1/300 dilution of specific anti-NS3 (Abcam, UK) for 1 hour at RT. Finally, cells were subjected to FITC-conjugated secondary antibody, washed by PBS and incubated with 1 µg/mL DAPI dye solution (Roche, Germany) and then, washed away by PBS. The stained cells were observed and imaged under Immunofluorescence microscope (BX52-Japan).

## 4. Results

### 4.1. Protein Expression and Western Blotting

Total proteins from cells transfected with three expression plasmids (wtNS3, muNS3 and NS3/4A), were analyzed using western blotting methods to evaluate the presence of NS3 protein.

**Figure 2.**
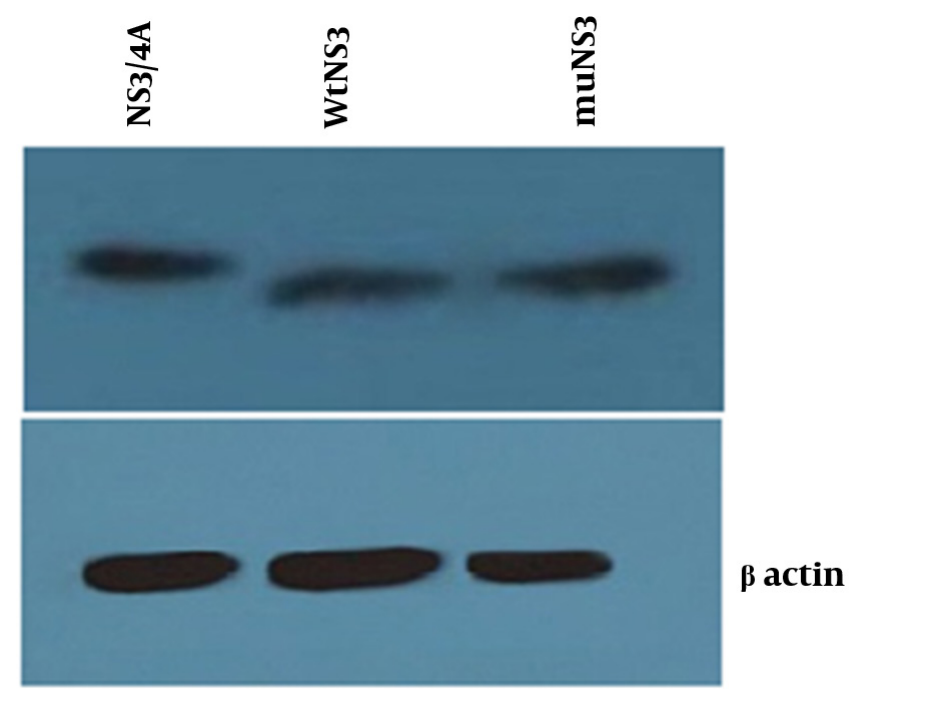
Confirmation of Constructs Expression Western blot test for confirming the expression of NS3/4A, wtNS3 and muNS3 (upper- from left to right) showed a similar rate of protein production by different plasmids, whereas -actin was used as an internal control (down). HepG2 cells were transfected with NS3/4A, wtNS3 and muNS3 and Western blot test was performed using polyclonal antibody against NS3.

**Figure 3.**
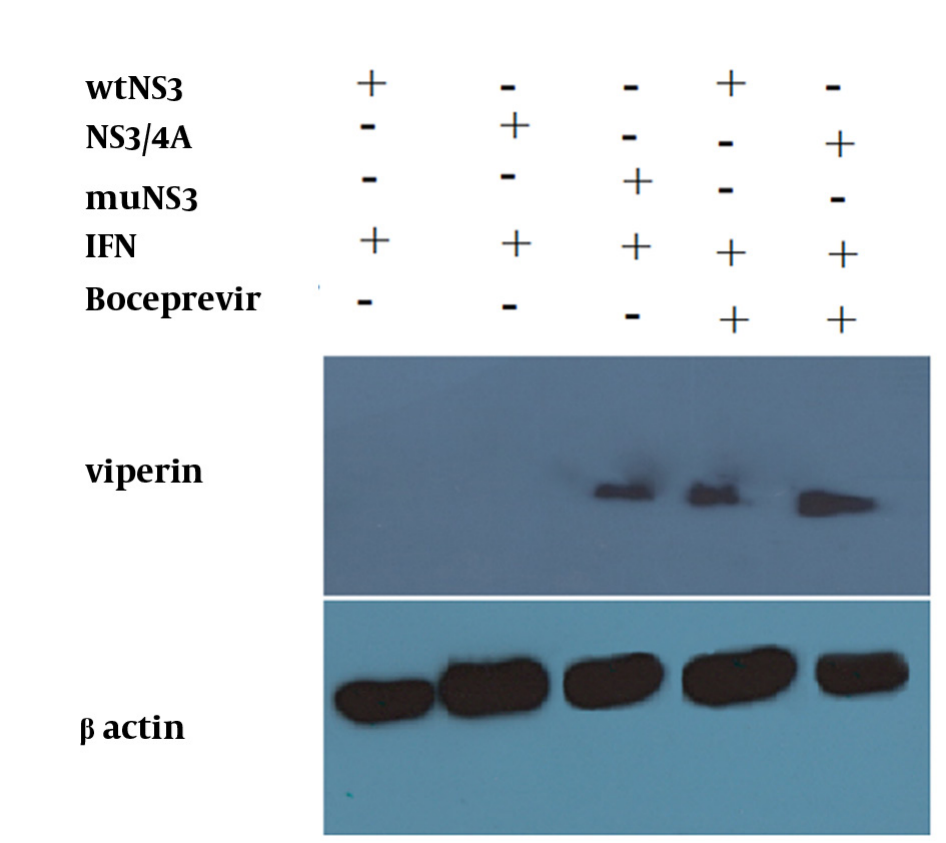
Expression of Viperin in the Presence of wtNS3, NS3/4A and muNS3 HepG2 cells were transfected with wtNS3, NS3/4A and muNS3 plasmids, and then treated with IFN-alpha. As showed here, wtNS3 and NS3/4A blocked the expression of viperin protein.

Neither mutations in the NS3 ser-139 nor presence of NS4A affected the expression of NS3. Besides, NS3 protein was observed as a 70 kD protein band in all of the samples (Figure 2). Expression of viperin was suppressed by wtNS3 and NS3/4A. Nonetheless, in the presence of muNS3 and in Boceprevir treated cells, viperin was observed as a 42 kD band in western blot test (Figure 3).

### 4.2. Quantitative PCR (qPCR)

**Figure 4.**
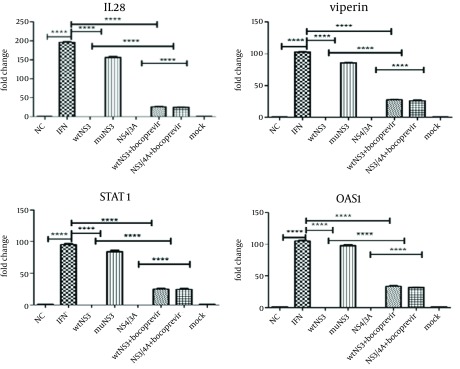
Expression of ISGs in Interferon Treated HepG2 Cells Cells were mock or IFN treated with 1000 IU/mL of IFN, 48 hours before, IFN treated cells were transfected with wtNS3, muNS3 and NS3/4A. After 6 hours, total RNAs were extracted and submitted to quantitative real-time PCR to detect the mRNAs of ISGs. A group of cells transfected with wtNS3 and NS3/4A, also was treated with Boceprevir . (****P<0.0001). NC, normal cell.

After optimization of newly developed real-Time PCR for different genes, this method was used for the expression analysis of viperin, OAS1, STAT1 and IL-28. In case of untreated cells (negative control group), the expression of selected genes was not detectable, while in the HepG2 cells treated with 1000 IU/mL of IFN-alpha expression of all mentioned genes was elevated up to a hundred fold. Here, previously recognized ISGs such as OAS1 and STAT1 were evaluated as controls due to their previously proven effects on IFN signaling pathways (15). Interestingly, data analysis on expressing genes showed that in the IFN treated cells, which were transfected with wtNS3 or NS3/4A plasmids, expression of viperin had been completely blocked. In parallel, expression genes analysis revealed that IL-28 and other control ISGs (OAS1 and STAT1) mRNA levels were significantly impaired in these groups. However, in the group transfected with muNS3 plasmid, expression of these genes remained elevated after IFN challenge due to lack of NS3 protease activity (Figure 4). Furthermore, for a detailed study of protease role in ISGs genes expression, in other cell groups which were first transfected with wtNS3 or NS3/4A, Boceprevir, a protease inhibitor agent which binds to ser-139 of NS3 active site (serine trap inhibitor) (20), was added to culture medium to transiently stop protease activity by NS3. The analysis of gene expressions in these groups also indicated that eventhough all genes expressions were detectable, they were significantly reduced to a lower rate, compared to the expression induction by IFN stimulated group as positive control and muNS3 plasmid transfected cells.

### 4.3. Immunofluorescence

**Figure 5.**
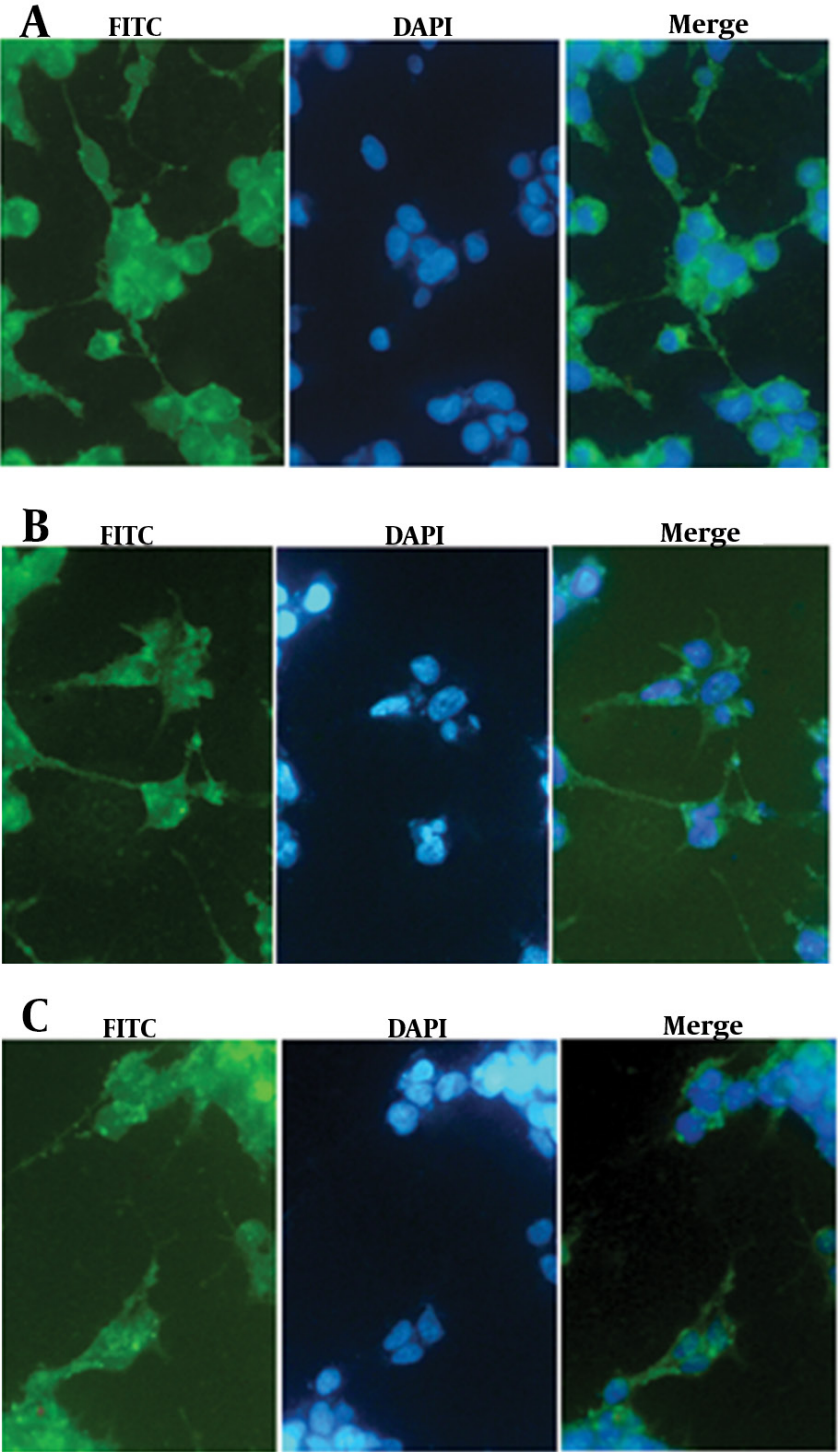
Immunofluorescent Staining Test for Transfected HEK293 Cells With Expressing Plasmid, NS3/4A (A), wtNS3 (B) and muNS3 (C). Distribution of NS3/4A is cytoplasmic whereas wtNS3 and muNS3 spread both in nuclear and cytoplasm. Pale green (yellowish shining) appearance is indicative of the co-presence of green (NS3 protein) and blue (nucleus) in wtNS3 and muNS3 transfected cells.

It is now accepted that NS3/4A is anchored on ER compartment because of an ER targeting peptide lied in NS4 protein (21). As shown, Immunofluorescence staining of the transfected cells revealed the distribution of NS3 protein among cell compartments. The staining results showed that free NS3 protein without NS4A anchoring protein is distributed both in cytoplasmic and nuclear compartments, as demonstrated in figure 5. In overlay fluorescent analysis of transfected cells, nucleus localized NS3 appeared in pale blue due to concomitant DAPI staining of nucleus while cytoplasm remained with sharp green shining. The presence of NS4A as an anchoring molecule led to NS3 association with ER and ER-like membrane, thus, inhibits NS3 translocation toward nucleus. DAPI stained nucleus is, then, distinguishable from green shining cytoplasm which had been enriched by NS3/4A (21) (Figure 5).

## 5. Discussion

The frontline of defense against viral infection is the IFN response, which triggers the induction of a wide range of antiviral proteins. Following the start of the IFN cascade, over 300 interferon-stimulated genes were induced (22). Viperin, an interferon-inducible protein, has been recently considered as a multifunctional protein with important antiviral activities (9). It has been previously shown that this protein disturbs HCV replication by interacting with NS5A (8, 23, 24). In cells infected with the virus, induction of viperin expression is boosted by both interferon-dependent and interferon-independent pathways. In the first pathway, extracellular IFNs is necessary whereas in the second one retinoic acid-inducible gene I (RIG-I)-like receptor (RLR) family sense viral double-stranded RNA then, reacts with MAVS which finally leads to type I IFNs and ISGs induction (8). In HCV infection, it is not clearly determined by which pathway viperin expression is affected more (13). It has been reported that NS3 protein disrupts the induction of ISGs in IFN response pathway (4, 25). A previous study showed that HCV proteins inhibit the Jak-STAT pathway (26). In our study, we showed that IFN signaling Jak-STAT pathway was interrupted after expression of this viral protein. Further studies are required to determine the mechanism of this interruption. The quantitative analysis of gene expression showed a significant blockade in viperin expression in the presence of NS3 protease activity both when fused with NS4A and in single form, while IFN induction increased viperin expression in comparison to that in the negative control cells. After treatment with Boceprevir in NS3 and NS3/4A transfected cells, also the viperin expression restarted. Furthermore, increased gene expression in Boceprevir treated group was similar in NS3 and NS3/4A transfected cells, which suggested a similar function. This experiment reestablished the effective potency of an anti-protease drug to boost the immune response through its action on NS3 protease activity. Moreover, both NS3/4A and free NS3 were evaluated as substrates for Boceprevir; a point not mentioned in previous studies and the results indicated that both forms of protein were targeted by the drug. Of course, further research is required to verify this finding. Our findings suggest that protease activity of NS3 abrogate the expression of viperin mRNA following IFN- induction. It is interesting to look for other mechanisms of direct disrupting of viperin expression or function by NS3 protease activity. Besides, in our study we tried to evaluate the expression of IL-28 (type III IFN) during IFN stimulation in the presence of NS3 protease activity. Surprisingly, the gene expression results showed that the expression of IL-28, which is also an important inducer of viperin, was modulated in the presence of functional NS3. Therefore, it could be concluded that IL-28 elevation after NS3 protease blockade by Boceprevir (as achieved here) ultimately induced more viperin expression in turn. This finding along with the fact that NS3 is able to disrupt other ISGs induction and IFN expression, demonstrates that HCV NS3 may affect the expression of viperin by several means.

To our knowledge, since most previous studies focused on NS3/4A complex as viral protease except one study that showed the free NS3 (without NS4A) affects overall IFN- response (25), it was interesting to investigate and confirm the effects of NS3 protease activity with or without NS4A help in more details. To do so, we evaluated the protease activity of NS3 single protein versus that of NS3/4A fusion protein and its final consequence on the expression of some ISG genes. Our findings again (25) emphasized the dispensable role of NS4A in immune modulatory role of NS3 at least for IFN-I pathway. The results showed that functional NS3 without or with NS4A blocked some ISGs expression. In addition, to delineate the importance of NS3 protease activity, we prepared a mutated NS3 construct with abolished protease activity (muNS3). By comparing mutated and wild type constructs, we showed that protease activity of NS3 plays a profound role in the disruption of Jak-STAT pathway. In the next study, using mu NS3/ 4A seems necessary. Meanwhile, to ensure similar protease activity of NS3 and original NS3/4A, we evaluated the expression of some ISGs like STAT1 and OAS1, controls, which are acclaimed to be down regulated by NS3/4A frequently (15). As expected, our results for ISGs expression analysis supported previous reports, indicating the important role of protease activity of NS3 but altogether a dispensable role for NS4A that was less addressed in previous studies (27, 28). In the groups of those cells which were first transfected with wtNS3 or NS3/4A and then, treated with Boceprevir, the level of expression of ISGs genes recovered, compared to that in the group not treated with antiprotease (P < 0.0001) due to protease blockade but was not equal to those observed in cells stimulated only by IFN. We speculated that this might be due to partial inhibition of protease activity by Boceprevir in such experiments. For subsequent studies, it would be interesting to evaluate the level of viperin early in infection among patients who clear HCV infection, and to determine whether it could be exploited as a predictive factor in clinical managements, like those explored for IL-28 (29). On the other hand, a critical mutation showed a profound role in affecting NS3 protease activity and subsequently in IFN response pathway, but it is not clear whether this mutation has direct impacts on carcinogenesis, fibrogenesis and miRNA induction pathways of gene expression. Further studies are needed to investigate how IFN response and respective inflammatory factors are influenced by NS3 protein.
